# Engineering of Active and Passive Loss in High-Quality-Factor Vanadium Dioxide-Based BIC Metasurfaces

**DOI:** 10.1021/acs.nanolett.4c01703

**Published:** 2024-08-27

**Authors:** Andreas Aigner, Filip Ligmajer, Katarína Rovenská, Jakub Holobrádek, Beáta Idesová, Stefan A. Maier, Andreas Tittl, Leonardo de S. Menezes

**Affiliations:** † Chair in Hybrid Nanosystems, Nano-Institute Munich, Faculty of Physics, Ludwig-Maximilians-University Munich, Munich 80539, Germany; ‡ Central European Institute of Technology, Brno University of Technology, 61200 Brno, Czech Republic; § Institute of Physical Engineering, Faculty of Mechanical Engineering, Brno University of Technology, 61669 Brno, Czech Republic; ∥ School of Physics and Astronomy, Monash University, Clayton, Victoria 3800, Australia; ⊥ Department of Physics, Imperial College London, London SW7 2AZ, United Kingdom; # Departamento de Física, Universidade Federal de Pernambuco, 50670-901 Recife, PE, Brazil

**Keywords:** Active metasurfaces, bound states in the continuum, vanadium dioxide, loss tunability, near-field tunability

## Abstract

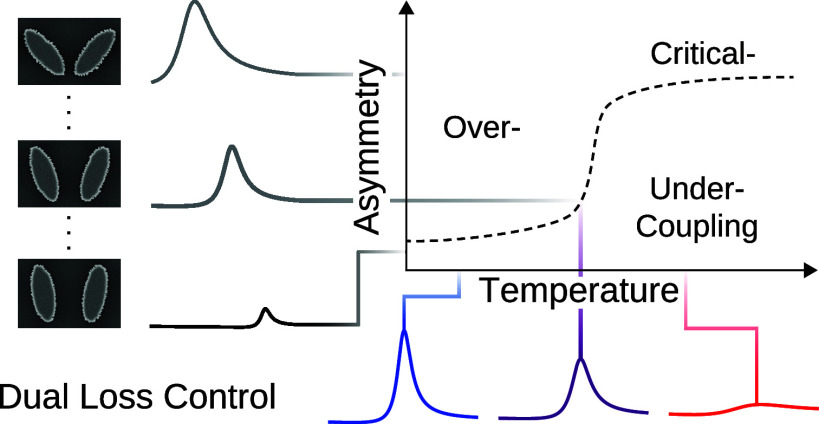

Active functionalities of metasurfaces are of growing interest in nanophotonics. The main strategy employed to date is spectral resonance tuning affecting predominantly the far-field response. However, this barely influences other essential resonance properties like near-field enhancement, signal modulation, quality factor, and absorbance, which are all vital for numerous applications. Here we introduce an active metasurface approach that combines temperature-tunable losses in vanadium dioxide with far-field coupling tunable symmetry-protected bound states in the continuum. This method enables exceptional precision in independently controlling both radiative and nonradiative losses. Consequently, it allows for the adjustment of both the far-field response and, notably, the near-field characteristics like local field enhancement and absorbance. We experimentally demonstrate continuous tuning from under- through critical- to overcoupling, achieving quality factors of 200 and a relative switching contrast of 78%. Our research marks a significant step toward highly tunable metasurfaces, controlling both near- and far-field properties.

Structuring matter on a subwavelength scale offers unparalleled control over spectral response,^1^ beam propagation,^2^ and localized electric fields.^3^ Metasurfaces,^4,5^ two-dimensional arrays of subwavelength resonators, have been at the forefront of this innovation enabling significant advancements across various photonic fields, including flat lenses,^2,6^ holograms,^7,8^ molecular sensors,^9−11^ lasers,^12,13^ and photocatalytic reactors.^14,15^ However, their predominantly passive nature severely limits their practical use. Consequently, the focus has shifted toward developing active solutions^16−18^ that manipulate the refractive index (RI) of materials through external stimuli like electrical tuning,^19,20^ optical illumination,^21−23^ or temperature.^24,25^

Besides achieving high switching contrasts for transmitted or reflected light in the far-field,^20,26^ it is crucial to finely tune resonance parameters like quality (Q) factor, field enhancement, and light absorbance within the material. This is especially important for applications relying on tailored resonance characteristics like photocatalysis, thermal emission, molecular sensing, or in systems sensitive to the interplay of different loss rates, particularly for polaritonic and exceptional-point metasurfaces.^27,28^ However, the widely used resonance detuning based switching falls short for these applications. There the resonance is merely shifted spectrally, without significantly impacting the other critical resonance parameters mentioned. Furthermore, the employed switching materials are typically lossy, making high Q-factor resonances challenging due to their vulnerability to material losses. This includes most well studied active materials like germanium–antimony-telluride (GST),^29,30^ indium–antimony-telluride,^22,31^ conductive polymers,^32,33^ and vanadium dioxide (VO_2_).^34,35^

Recently, symmetry-protected bound states in the continuum (BICs)^36,37^ have gained attention in the metasurface community. Their appeal lies in their spectrally narrow resonances quantified by the Q-factor, which is the ratio of resonance frequency ω_0_ to resonance bandwidth *δω*, and in the straightforward tunability of far-field coupling through simple geometric parameters. This led to demonstrations of numerous exciting physical phenomena with applications for lasers,^38^ chiral resonators,^39^ molecular sensors,^40^ color printing,^41^ and flat lenses.^42^ Applying the principle of BICs to tunable metasurfaces is, however, experimentally challenging. Popular tunable materials like GST, conductive polymers, or VO_2_ have not yet been achieved experimentally due to the susceptibility of high Q-factor resonances to intrinsic losses.^43^ Also, first active BIC metasurfaces have achieved remarkable results, including almost unitary circular dichroism switching for chiral applications,^44^ ultrafast switching times of <2.5 ps accompanied by a switching contrast of 0.3,^23^ and active modulation of wavefronts.^24^ However, to date, active BICs have been primarily limited to low refractive index changing materials^23,44,45^ or to the terahertz range.^46^

Here, we propose a new approach for active fine-tunable metasurfaces based on loss-engineered VO_2_-Si resonators^47−49^ and BIC resonances. Rather than fighting the high intrinsic losses in the high-temperature phase of VO_2_,^35^ we utilize them for loss-based active tuning. Because the temperature induced phase change in VO_2_ starts locally in subwavelength areas,^50^ the overall phase transition is gradual, with intermediate states readily accessible within a temperature window of roughly 30 °C. When combined with the BIC’s asymmetry-based tunability of radiative losses, this approach provides two tuning mechanisms (Figure 1a): a passive one via the asymmetry of the structure (Figure 1b) and an active one via the temperature (Figure 1c). This dual control allows to precisely tailor various resonance parameters like Q-factor, resonance amplitude, and local field enhancement. Our numerical and experimental demonstrations reveal that for a conventional, ellipse-pair based BIC metasurface, a thin VO_2_ layer of about 30 nm is sufficient to quench the resonance. The remaining 720 nm resonator height can be comprised of any low-loss dielectric. In our case, 600 nm of silicon are below and 120 nm of SiO_2_ are on top of the VO_2_ layer (see a sketch of the structure in Figure 1d and the scanning electron microscope (SEM) image in Figure 1e). With VO_2_ in the low-temperature phase, we achieve Q-factors of up to 200 and a maximum reflectance amplitude of 90%. This represent a significant advancement over previous VO_2_-based active metasurfaces, surpassing their Q-factors by an order of magnitude, while still maintaining a competitive switching contrast, see comparative Table S1. By heating the metasurfaces gradually we can finely tune the reflectance amplitude (Figure 1f) and achieve a relative switching contrast of up to 78% in experiments (Figure 1g,h). We map the parameter space defined by the asymmetry parameter α and temperature *T*, which govern radiative and intrinsic losses, respectively. Using temporal coupled mode theory (TCMT) modeling, we identify the impact of our dual-control mechanism on both loss rates, and thus also on the Q-factors and absorbance. Additionally, our results confirm the structure’s versatility in transitioning between all coupling states, ranging from under- through critical- to overcoupling.

**Figure 1:**
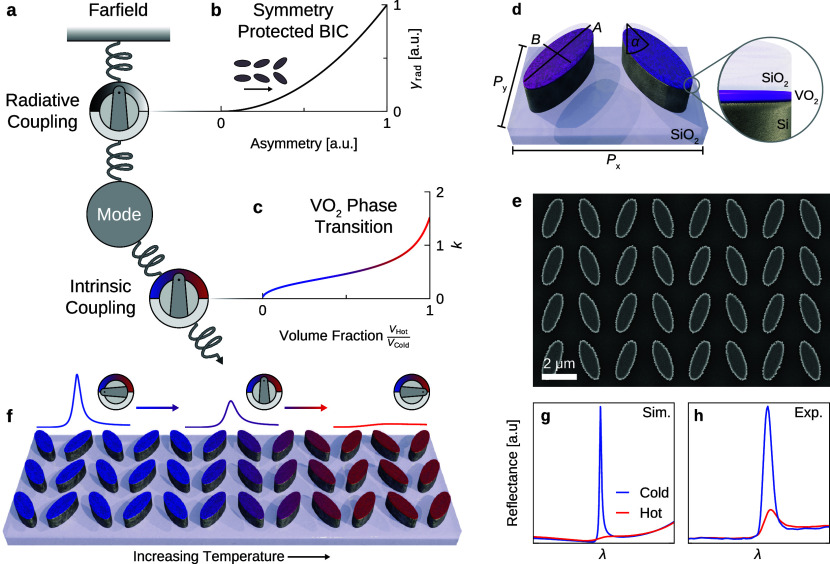
**Tuning principle and resonator geometry**. (a) Schematics of the active VO_2_-Si BIC metasurface. The resonator is coupled to incoming and outgoing radiation via asymmetry-dependent radiative loss γ_rad_. γ_rad_ ∝ sin^2^(α) with α as the tilting angle of the dual ellipse geometry in (b). Additionally, the mode is coupled to intrinsic losses which can be fine-tuned by the gradual phase transition of VO_2_ and its increasing imaginary part *k* of the complex RI in (c). (d) Sketch of the tilted ellipse-pair unit cell defined by the ellipses’ long axis *A*, the short axis *B*, the tilting angle α, and the pitch in the *x*-direction *P*_*x*_ and in the *y*-direction *P*_*y*_. The resonator, on top of a SiO_2_ substrate, consists of a Si layer, a VO_2_ layer, and a capping SiO_2_ layer. (e) SEM image of the fabricated metasurface with α = 20°. (f) Sketch of the metasurface with a gradual phase transition from cold to hot indicated by the color transition from blue to red and the thermostat icons. Sketched (g) numerical and (h) experimental reflectance spectra for the VO_2_-Si metasurface in its hot and cold phases.

We chose a tilted ellipse-pair geometry for our metasurface design due to its remarkable resonance robustness.^51^ Furthermore, compared to other designs, this geometry offers a notably low spectral reflectance baseline of 2–3% in simulations. This feature, along with an exceptionally clear off-resonant spectrum, makes it particularly suitable for switching applications that value pristine spectra, high switching contrasts, and multiplexing capabilities. While we focus on the tilted ellipse pair, it is worth noting that our methodology is not constrained to this specific BIC metasurface geometry and can be applied to other geometries.

To implement our loss-based switching concept, we need an active material that satisfies two main requirements. In its “off” state, the losses of the material should be minimal to ensure that resonant modes are not damped. Conversely, in its “on” state, the material should have a very high absorbance to achieve optimal resonance quenching. VO_2_, especially in the mid-IR, stands out as an extraordinary choice, meeting both criteria.^34,35^ Our atomic layer deposited VO_2_ (see Methods in Supporting Information) exhibits at 6.5 μm an extinction coefficient *k* of 0.011 at 25 °C, and a *k* of 2.42 at 80 °C, as validated via ellipsometry (see Figure S1).

Using simulations (see Methods in Supporting Information), we tuned the geometric parameters of our structures to resonate between 6 and 7 μm. This leads to a 750 nm high resonator on top of a SiO_2_ substrate, which is composed of a Si layer at the bottom, a VO_2_ layer in the middle, and a 120 nm SiO_2_ capping layer which acts as a mask during the VO_2_-Si etching. We chose to position the VO_2_ layer in the top part of the resonator to ensure high coupling with the resonant mode and straightforward fabrication. Simulations with a continuous VO_2_ film below the resonators (Figure S4) show good switching performance but increased off-resonance absorbance, especially for higher layer thickness needed as the overall coupling in this configuration is lower. While SiO_2_ has some absorption in the mid-IR range, it was chosen as substrate material since its thermal expansion coefficient aligns well with Si, ensuring a stable film during the VO_2_ annealing process. We sourced the RIs for VO_2_ through ellipsometry, shown in Figure S1, and took the values for Si and SiO_2_ from Shkondin et al.^52^ and Kischkat et al.,^53^ respectively. To model the intermediate states of the VO_2_ layer, where it is partially in the cold and partially in the hot phase, we employed an effective medium approximation^54^ (Supporting Note 1) with a hot-phase volume fraction *VF* = *V*_hot_/*V*_total_, defined as the ratio of volume in the hot phase *V*_hot_ divided by the total volume *V*_total_.

Resonance detuning-based switching typically requires a resonator predominantly composed of an active medium, because it changes the effective cavity size through refractive index shifts. Conversely, our loss-based approach only needs a minimal portion of the medium to be active. With this method, the effective cavity size hardly changes, instead, we merely introduce intrinsic losses into the system to attenuate the mode. By examining the relationship between the ellipses’ tilting angle α, the VO_2_ layer height, and the resulting reflectance modulation, we observed absolute reflectance modulation amplitudes max(*R*_cold_-*R*_hot_) of up to 80% (see Figure 2a). Note that this corresponds to a maximum relative modulation max[(*R*_cold_ – *R*_hot_)/*R*_cold_] of 97% (Figure S2). Notably, as the asymmetry increases, the reflectance modulation peaks between α = 15–30°, after which it declines. We ascribe this trend to two counteracting effects: the resonance lifetime and the absorption of VO_2_ in its cold state. At smaller asymmetries with high Q-factors *Q*, the longer resonance lifetime (given by τ = *Q*/ω_0_) allows light to be in extended contact with the VO_2_ layer and thereby the absorption is enhanced. However, VO_2_ also exhibits absorption when in its cold state (*k* = 0.011 at 6.5 μm), which results in a lower overall reflectance amplitude for small asymmetries in the cold phase. Thus, thin VO_2_ layers achieve optimal modulation with small asymmetries, and thicker layers favor larger asymmetries. Due to the dampening influence of a thicker VO_2_ layer on both the Q-factor and the reflectance amplitude in the cold phase, we proceed with a 30 nm VO_2_ layer for the remainder of the manuscript, which allows for both high Q-factors and strong resonance modulation. In Figure 2b, the absolute reflectance modulation across various asymmetries is depicted. It underlines the spectral cleanliness of the switching behavior since no modes other than the BIC are present, and thanks to the thin VO_2_ layer, we can switch the metasurface with no noticeable spectral detuning and no off-resonance modulation (Figure 2b).

**Figure 2:**
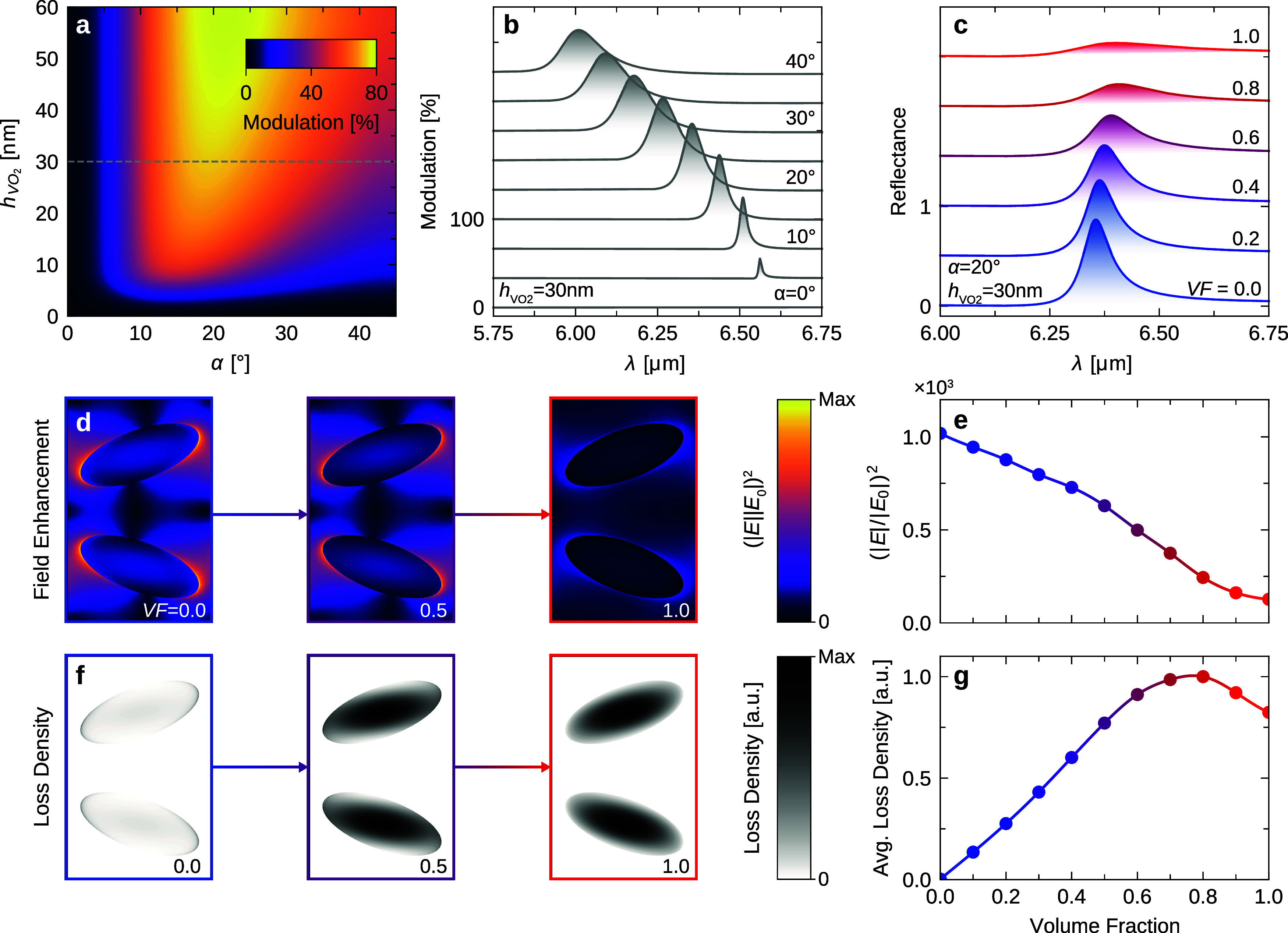
**Numerical studies on the switching behavior**. (a) Absolute reflectance modulation max(*R*_cold_ – *R*_hot_) around 6.0–6.5 μm as a function of the ellipse tilting angle α and the VO_2_ layer height *h*_VO_2__. A dashed gray line indicates *h*_VO_2__ = 30 nm, presented in (b) where the spectral difference between cold and hot phase reflectance is visualized for various asymmetries, with a vertical offset of 0.33 between the spectra for clarity. (c) Spectra of different switching states (different *VF*) for *h*_VO_2__ = 30 nm and α = 20°. Spectra are vertically shifted by 0.5 for clarity. (d) Field enhancements in the VO_2_ layer in the cold (*VF* = 0.0), intermediate (*VF* = 0.5), and hot (*VF* = 1.0) phases for the same geometric parameters as in (c). (e) Maximal field enhancement for different *VF*. (f) Loss density mapping analog to (d). (g) Average loss density for different *VF*.

We expand our numerical analysis beyond the search for maximum switching in order to study the intermediate switching states. Figure 2c captures the progressive degradation of the resonance with increasing *VF* for α = 20°. It reveals a gradual decrease in both Q-factor and amplitude as *VF* increases, until the resonance almost vanishes. To study the gradual quenching of the BIC in more detail, Figure 2d presents simulated maps of the electric field enhancement (FE) within the VO_2_ layer for varying *VF*. For *VF* = 0.0 (cold phase), there are pronounced FE hotspots at the tips of the ellipses (*FE* = 1019), which reveal the well-known electric dipole profile in each of the ellipses that form this BIC mode. While *VF* increases, the field configuration remains consistent, but the FE eventually drops by almost an order of magnitude at *VF* = 1.0 (hot phase, *FE* = 126). Figure 2e depicts the maximal FE extracted from the simulations across all *VF* in increments of 0.1, demonstrating an almost linear decline. To offer further insight into the attenuation of the mode, we present simulated loss density maps (power absorbed per unit volume^55^) for the varying *VF* in Figure 2f. At *VF* = 0.0, losses manifest in regions with high FE (the tips of the ellipses, consistent with Figure 2d), a typical behavior of resonant structures. In contrast, for *VF* = 1.0, the losses intensify and disperse, peaking in the centers of the ellipses, which indicates nonresonant absorption. The intermediate state with *VF* = 0.5 is a combination of cold and hot phase characteristics with losses both in the center and at the tips. As shown in Figure 2g, the average loss density within the VO_2_ layer increases most rapidly for *VF* between 0.2 and 0.5. A higher *VF* lead to a saturated loss level, until it declines for *VF* = 0.9–1.0, possibly due to the further decreasing FE.

Having verified through simulations that a thin VO_2_ layer within a BIC resonator can dampen the resonance efficiently and in a controlled manner, we next focus on experimental verification of this effect. We fabricated the BIC metasurface as detailed in the Methods section (Supporting Information). All optical measurements were conducted using a Spero microscope (Daylight Solutions), which operates in the wavelength range starting from 5.6 μm, as described in the Methods section (Supporting Information). The upper wavelength limit was set to 7.5 μm because, at longer wavelengths, the SiO_2_ phonon band of the substrate and the capping layer dominates the optical response. Aiming to demonstrate the independent tuning of both intrinsic and radiative losses, we fabricated metasurfaces with varying asymmetries, ranging from α = 0° to α = 40° in the increments of 5°. Figure 3a presents their reflectance spectra measured at 30 °C (*R*_cold_) and 100 °C (*R*_hot_), from which we extracted their absolute reflectance modulation characteristics (*R*_cold_ – *R*_hot_) in Figure 3b. Notably, the maximum modulation varies with the asymmetry, reaching almost 50% at α = 20°. Generally, the switching process does not compromise the clean low-temperature spectra and produces only minimal off-resonance wavelength modulation. The broadening of the mode at high temperatures is relatively small, while the Q-factor of the reflectance modulation is on par with the resonance in its cold phase. The off-resonance modulation observed around 5.8 μm for α = 40° arises from the BIC interacting with the onset of the first diffraction order at 5.7 μm.

**Figure 3:**
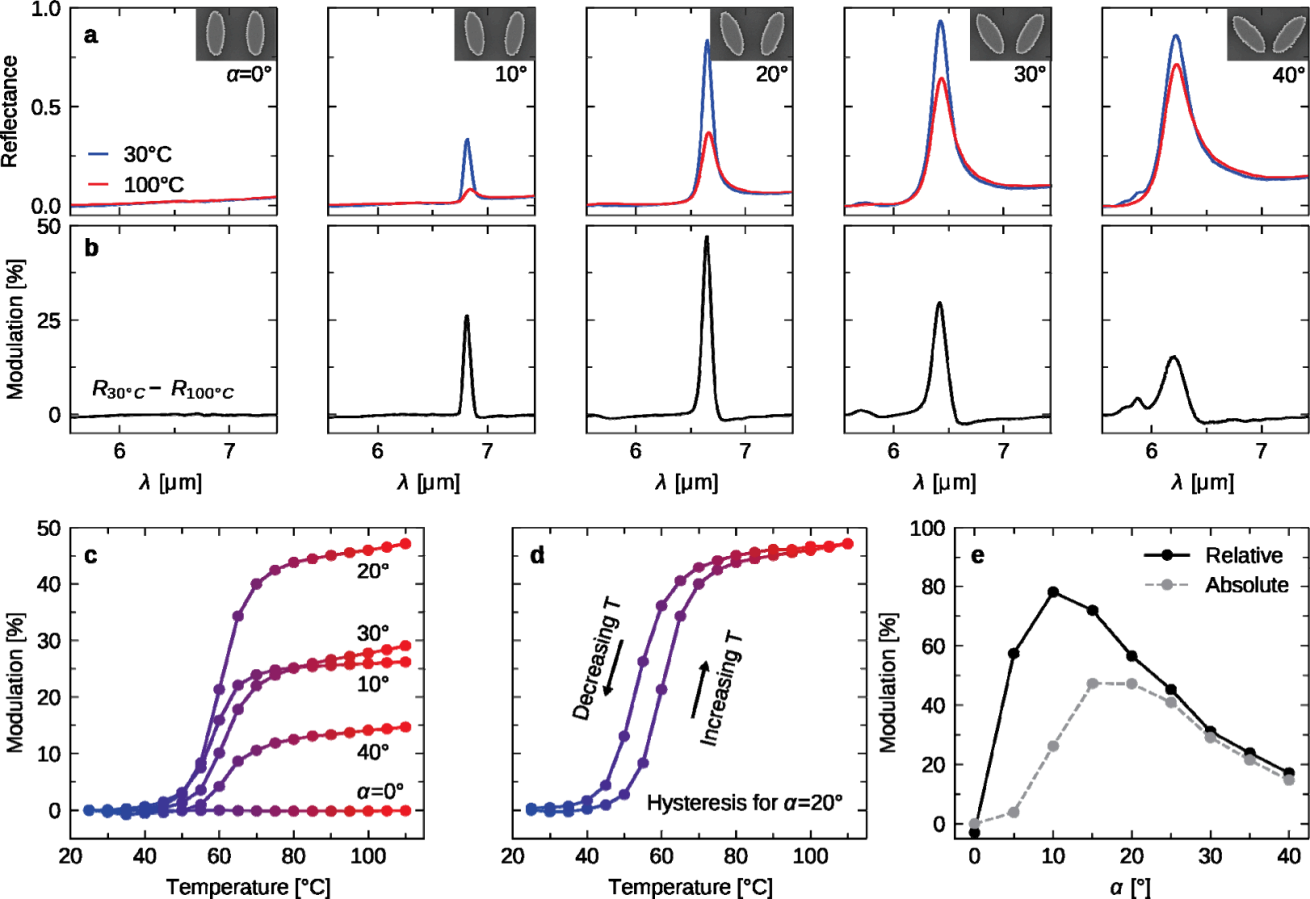
**Experimental switching verification**. (a) Measured reflectance spectra at 30 °C (blue) and 100 °C (red) for increasing α. Insets in the top right corners show SEM images of the respective unit cell with the dimensions of 4400 × 2860 nm^2^. (b) Reflectance modulation (*R*_30°C_ – *R*_100°C_) extracted from the spectra in (a). (c) Temperature-dependent variation of the absolute reflectance modulation max(*R*_30°C_ – *R*_100°C_) for the same asymmetries as in (a) and (b). (d) Full hysteresis modulation pattern for α = 20°. (e) Absolute max(*R*_30°C_ – *R*_100°C_) and relative reflectance modulation max[(*R*_cold_ – *R*_hot_)/*R*_cold_] across all asymmetries.

To assess the fine-tuning capabilities of our metasurfaces, we varied the temperature from 25 to 110 °C in 5 °C increments. The absolute reflectance modulation amplitude max(*R*_cold_ – *R*_hot_) across different asymmetries is plotted in Figure 3c. The phase transition occurs between 45 and 75 °C, with the most significant changes around 60 °C. This offers a sizable 30 °C temperature window to fine-tune the optical response. In line with the spectra in Figure 3a,b, the highest maximal modulation is achieved with α = 20°, while 10° and 30° show similar results. An asymmetry of 40° yields much lower modulation, and 0° shows negligible change. Driving the metasurface through the whole modulation cycle by heating and cooling reveals a typical hysteresis, as expected for VO_2_-modulated systems.^34^ Our dual-tuning approach lets users prioritize either the *absolute* reflectance modulation discussed so far or the *relative* reflectance modulation max[(*R*_cold_ – *R*_hot_)/*R*_cold_]. Based on this preference, the optimal structural asymmetry can be chosen, with the peaks for relative and absolute reflectance modulation in our presented structure occurring at α = 10*°* and α = 20°, respectively, see Figure 3e.

Having demonstrated the experimental switching and fine-tuning capabilities of our metasurfaces, we now take a closer look into the effects the switching has on the resonance characteristics. Our investigation involves spectra from eight distinct asymmetries (α = 5–40° in 5° increments) and a temperature series comprising 18 steps (*T* = 25–110 °C in 5 °C increments). This results in a 2D parameter matrix of 144 unique resonance spectra. To model these resonances, we used temporal coupled mode theory (TCMT), an established framework for interpreting complex light-metasurface interactions.^56,57^ Central to TCMT are intrinsic γ_int_ and radiative γ_rad_ loss rates, signifying energy loss within the resonator and energy loss to external channels, respectively. Controlling their ratio is crucial since they dictate the Q-factor, control the FE, and determine the resonance behavior. In our modeling, we conceptualize our system as a singular resonator coupled to two ports, one symbolizing incoming/reflected light and the other indicating transmitted light, as detailed in Supporting Note 2. Next, we fit the TCMT model to our experimental data by using unconstrained parameters. Only the intrinsic loss γ_int_ is constrained for all α at a given temperature since it predominantly depends on the intrinsic loss of VO_2_. In Figure 4a, we replot the experimental reflectance data from Figure 3a (*T* = 25 °C, varying α, solid gray lines) and overlay them with the results of the TCMT fit (dashed black lines).

**Figure 4:**
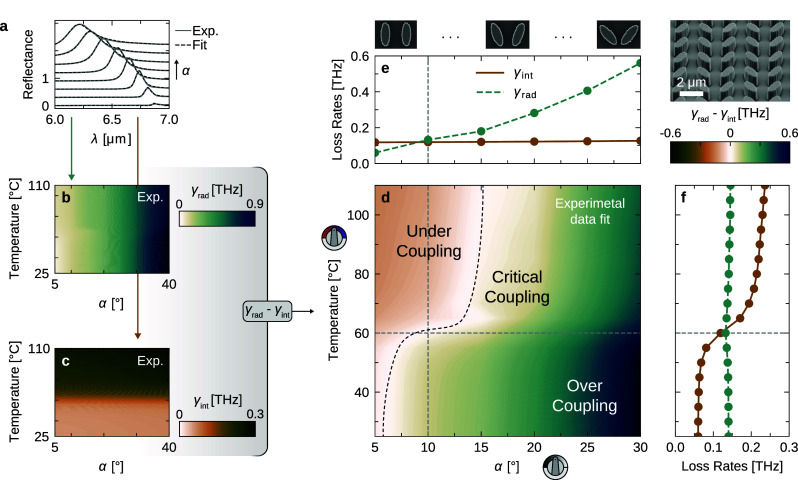
**Insight into experimental data through TCMT analysis: (a)** Measured spectra (gray solid line and shading) and TCMT-derived fits (dashed black line) for metasurfaces at 25 °C with α values from 5° to 40°. (b) Radiative loss γ_rad_ and (c) intrinsic loss γ_int_ extracted from TCMT fits of experimental data across various asymmetries α and temperatures. While γ_rad_ exhibits an increase with α and is constant over temperature, γ_int_ behaves oppositely: it increases with temperature and is unaffected by α. (d) Overlay of data from (b) and (c) displaying the difference between the two loss rates γ_rad_ – γ_int_. Brown shading marks undercoupled resonator regions (γ_rad_ < γ_int_), green denotes overcoupled regions (γ_rad_ > γ_int_), and white represents critically coupled resonators, indicated by the s-shaped dashed black curve. (e) γ_rad_ and γ_int_ plotted as functions of the angle α at a temperature of 60 °C, based on the data slice highlighted by the dashed horizontal line in (d). (f) γ_rad_ and γ_int_ plotted against temperature for α = 10°, corresponding to the data slice marked by the dashed vertical line in (d).

We extracted the loss rates γ_rad_ (Figure 4b) and γ_int_ (Figure 4c) as well as the corresponding total Q-factor (Figure S3) from all spectra and mapped them across the entire α-*T*-parameter space. As γ_rad_ represents the energy radiated out of the resonator, its expected rise with increasing asymmetry is evident, with no dependence on temperature. Conversely, γ_int_, which represents scattering and material losses, exhibits a substantial change with temperature around the phase transition of VO_2_. Analyzing these loss rates individually underlines a central observation: the two “tuning knobs” (asymmetry and temperature) modulate the two loss channels (γ_rad_ and γ_int_) independently, allowing selective tailoring of the resonance characteristics. Of special importance is the regime of critical coupling, where γ_rad_ equals γ_int_, marking a sweet spot for a plethora of photonic devices.^14,58,59^ It allows optimal energy transfer, enhances sensitivity, and maximizes light absorption. Figure 4d illustrates the interplay of the two loss rates within our parameter space by plotting their difference γ_rad_ – γ_int_. The brown zone corresponds to undercoupling (γ_rad_ < γ_int_), mainly observed in regions with high γ_int_ (elevated temperatures) and low γ_rad_ (minimal asymmetry). Overcoupling (γ_rad_ > γ_int_), on the other hand, is prevalent at lower temperatures and high asymmetries. The s-shaped dashed black curve marks the domain where resonators are critically coupled.

Our method of integrating a γ_rad_-adjustable BIC with the dynamically tunable γ_int_ of VO_2_ allows our metasurfaces to modulate between under, over, and critical coupling, dependent on temperature (shown in Figure 4e for *T* = 60 °C) or asymmetry (depicted in Figure 4f for α = 10°). A single metasurface can thus be effortlessly tuned through all coupling regimes, with the steepest changes within the phase transition temperature range of 45 to 75 °C. The path of critical coupling within our 2D parameter space can be further adjusted by simply adjusting the height of the VO_2_ layer (see Figure 2a).

Our study introduces a novel method for active control of metasurfaces by harnessing loss-based tuning within a phase-change material, specifically VO_2_, in a hybrid BIC metasurface. This approach combines the passive radiative loss control of BICs with the active intrinsic loss tunability of VO_2_. We achieved a numerical absolute reflectance modulation of 80% and a relative modulation of 97%. In experiments, these values were realized as 47% and 78%, respectively. Our method proves particularly effective for high Q-factors, resulting in a pristine reflectance spectrum free from off-resonant modulations and spectral clutter. We further experimentally verify the fine-tuning capability of our metasurfaces, which can be attributed to the broad hysteresis of VO_2_.

Besides far-field tuning, our design allows to modulate both near-field enhancement and absorbance through temperature variations. Moreover, experimental evidence shows the independent tunability of the system’s primary loss mechanisms: the radiative loss, passively controlled by the asymmetry factor of our geometry, and the intrinsic loss, tunable both actively via temperature and passively through the layer thickness of VO_2_. Using TCMT modeling we show how our metasurfaces can be tuned across all coupling states, from under- through critical- to overcoupling. Particularly, systems that depend on precisely adjusted loss rates, such as those of strongly coupled polaritonic or exceptional-point metasurfaces, benefit significantly from this high degree of tunability.

While our study focused on the mid-IR range, the underlying design principles are transferrable to the near-IR by adjusting geometric parameters, as demonstrated in Figure S5. This spectral tunability opens doors to various applications, such as transmission modulators for telecommunication. Furthermore, our approach is adaptable to many unit cell configurations, only requiring spatial overlap between the BIC mode and the VO_2_ material. Placing the VO_2_ layer close to the electric field hotspots can enhance the coupling and minimize off-resonance switching. We believe that our demonstrated dual-control mechanism can be applied to a wide range of material systems. These include switchable polymers^26^ and GST,^29^ as well as optically pumped materials like silicon.^46^

Our research provides significant insights into loss-based metasurface tuning, offering possibilities for applications where precise control of resonance parameters such as Q-factor, field enhancement, and absorbance is critical. These advancements pave the way for optical analog switches, adjustable narrow band transmitters and reflectors, and brightness tunable color-metasurfaces.

## Supporting Information Available

Methods section includes details on the fabrication workflow, including growth parameters for the Silicon film and VO_2_ film, as well as further patterning and etching parameters. It also describes the optical setup used for all far-field measurements. Additionally, there are sections on the effective medium approximation used and the TCMT model. A table comparing literature and further figures showing the permittivity of the materials and additional simulation results are also provided. (PDF)
