# Supplementary material for: Engineering of Active and Passive Loss in High-Quality-Factor Vanadium Dioxide-Based BIC Metasurfaces
Source: Nano Lett. 2024 Aug 27;24(35):10742–9. doi: 10.1021/acs.nanolett.4c01703 (PMC11389864; doi:10.1021/acs.nanolett.4c01703)
Supplement: Supplementary file 1 — nl4c01703_si_001.pdf [file nl4c01703_si_001.pdf]

Supporting Information for Engineering of Active and Passive Loss in High-Quality-Factor Vanadium Dioxide-Based BIC Metasurfaces

Andreas Aigner[1], Filip Ligmajer[2,3,], Katarína Rovenská[2,3], Jakub Holobrádek[2], Beáta Idesová[2,3], Stefan A. Maier[4,5], Andreas Tittl[1,*], and Leonardo de S. Menezes[1,6]*

1) Chair in Hybrid Nanosystems, Nano-Institute Munich, Faculty of Physics, Ludwig-Maximilians-University Munich, 80539 Munich, Germany.
2) Central European Institute of Technology, Brno University of Technology, 61200 Brno, Czech Republic.
3) Institute of Physical Engineering, Faculty of Mechanical Engineering, Brno University of Technology, 61669 Brno, Czech Republic.
4) School of Physics and Astronomy, Monash University, Clayton, Victoria 3800, Australia.
5) Department of Physics, Imperial College London, London SW7 2AZ, United Kingdom.
6) Departamento de Física, Universidade Federal de Pernambuco, 50670-901 Recife-PE, Brazil.

*E-mails: Filip.Ligmajer@vutbr.cz; andreas.tittl@physik.uni-muenchen.de

METHODS

Simulations for our study were performed using the commercial finite element solver CST Studio Suite (Simulia). The software was configured with adaptive mesh refinement, periodic boundary conditions, and operated in the frequency domain.

For sample fabrication, first, 600 nm of Si were deposited onto a fused silica substrate by an ion beam sputtering system with Kaufmann ion sources (Kaufman & Robinson, Inc.) at room temperature by using argon ions at 600 eV. Then, 30 nm of VO_2 were deposited by 250 cycles atomic layer deposition (Cambridge NanoTech Fiji 200) running at 150°C. Tetrakis(dimethylamino)vanadium (TDMAV) pre-heated to 87°C and water were used as precursors, that were let into the chamber within 0.6 s pulses with the consecutive waiting times of 8 s and 3 s, respectively. Immediately after the deposition, the sample was annealed in a vacuum tube furnace at 500°C for 5 minutes under 15 sccm flow of oxygen. Finally, 120 nm of SiO_2 for the etch mask were deposited onto the sample from a stoichiometric powder in an electron beam evaporator (Bestec, 8 kV, 1 Å/s) at room temperature. Note that the resulting VO_2 films exhibited slightly lowered phase transition temperature (60°C instead of conventional 68°C). We ascribe this effect to weak unintentional doping by hafnium atoms[17], most probably from the walls of our ALD chamber given it is often used for depositions of HfO_2. This notion is supported by TEM EDX analysis of our films.

Nanostructuring of the Si – VO_2 – SiO_2 multilayer film starts with a deposition of a 400 nm layer of positive electron beam resist, ZEP520A (Zeon Corporation), followed by a coating of a conductive polymer, using ESPACER (Showa Denko K.K). Electron beam lithography was carried out at 20 kV using an eLINE Plus system (Raith). Development was achieved with a subsequent bath in amyl acetate and MIBK:IPA in a 1:9 ratio. The polymer mask was used to dry-etch the SiO_2 layer and then removed by the Microposit Remover 1165 (Microresist). A second, selective dry-etching process based on SF_6 and Argon was applied to etch the VO_2 and Si layers. The ellipse's long axis A measures 2460 nm, the short axis B is 980 nm, the x-direction pitch P_x is 4400 nm, and in the y-direction pitch P_y is 2860 nm.

Optical measurements were conducted using a spectral imaging MIR microscope, Spero (Daylight Solutions). The microscope featured a 4× magnification objective ($NA = 0.15$) and provided a 2 mm^2 field of view. This system features three tunable quantum cascade lasers that consistently cover the 5.6-10.5 μm wavelength range, offering a spectral resolution of 2 cm^{-1}. The lasers emit linearly polarized light. Since the SiO_2 substrate restricts transmission, all measurements were carried out in reflection mode. To regulate the sample's temperature during experiments, a home-made sample holder was used. This holder consists of a thermally insulated copper contact surface, a temperature sensor, and four heating resistors. Temperature adjustments were controlled by a feedback loop to ensure stability.

SUPPORTING NOTES

Supporting Note 1: Effective Medium Approximation

To model the inhomogeneous nature of the intermediate state of VO_2 in our metasurfaces, we utilize the effective medium approximation which is widely adopted in literature[1,2,3]. It calculates the average permittivity of the composite material consisting of domains of VO_2 in its hot and cold phases and treats it as a homogeneous medium. This approximation is justified due to the highly subwavelength size of the domains in different phases within the polycrystalline film. The effective permittivity ε_{eff} of the VO_2 layer can be expressed as a weighted sum of the permittivities of its individual constituents, given by

$$\varepsilon_{\text{eff}} = VF \cdot \varepsilon_{\text{VO}_2,\text{hot}} + (1 - VF) \cdot \varepsilon_{\text{VO}_2,\text{cold}}$$

As the VO_2 undergoes the phase transition, the volume fraction VF, defined as $VF = \frac{V_{\text{hot}}}{V_{\text{total}}}$ varies, leading to changes in the effective permittivity ε_{eff}, see Figure S4. By integrating this effective medium approximation-based permittivity into our models, we could simulate the metasurfaces behaviour in intermediate states more accurately.

Supporting Note 2: TCMT Model

TCMT provides a straightforward method of modelling resonant structures. Fan et al. presents a general description[4], which we adapt for our single resonance system, coupled to two ports corresponding to reflectance and transmittance. The temporal dynamics of the resonance amplitude a can be written as

$$\frac{\mathrm{d}a}{\mathrm{d}t} = \left(-\frac{\gamma_{\mathrm{rad}} + \gamma_{\mathrm{int}}}{2} + i\omega_0\right) a + \sqrt{\gamma_{\mathrm{rad}}} s_{\mathrm{in}} \tag{1}$$

with ω_0 as the resonance frequency of the BIC. s_{in} represents the time dependent input field. Next, we define the relationship between the scattered field s_{out} with the resonance mode and the incoming field by

$$s_{\mathrm{out}} = \begin{bmatrix} s_{\mathrm{reflected}} \\ s_{\mathrm{transmitted}} \end{bmatrix} = S \begin{bmatrix} a \\ s_{\mathrm{in}} \end{bmatrix}$$

where S represents the scattering matrix given by

$$S = \begin{bmatrix} -i\sqrt{\gamma_{\mathrm{rad}}} & 0 \\ 0 & i\sqrt{\gamma_{\mathrm{rad}}} \end{bmatrix}.$$

By using equation (1) under steady state conditions, we are now able to calculate the reflectance R and the transmittance T via their relation to $s_{\mathrm{reflected}}$ and $s_{\mathrm{transmitted}}$:

$$R = |s_{\mathrm{reflected}}|^2 = \gamma_{\mathrm{rad}} |a|^2 = \gamma_{\mathrm{rad}} \left| \frac{\kappa}{i\omega + \frac{\gamma_{\mathrm{rad}} + \gamma_{\mathrm{int}}}{2}} \right|^2 |s_{\mathrm{in}}|^2$$

$$T = |s_{\mathrm{transmitted}}|^2 = \gamma_{\mathrm{rad}} |s_{\mathrm{in}}|^2$$

κ denotes the coupling coefficient between the resonant mode and the external ports. We used this theoretical framework to fit our experimental reflectance spectra to extract parameters like γ_{rad} and γ_{int}. For fitting purposes we normalized s_{in} to 1.

Resonator system	Resonance type	Spectral range	Exp. Q-factor	Switching contrast	Reference
Tunable Mie-resonant dielectric metasurfaces	Mie	Near-IR	14	0.2 (Transmittance)	[18]
Active metasurface based on Si/VO_2 hybrid meta-atoms	Mie	Mid-IR	<15	0.54 (Transmittance)	[19]
Hybrid metal dielectric metasurface	Plasmonic	Mid-IR	13	0.5 (Reflectance)	[20]
Electrically tunable metal metasurface	Plasmonic	Mid-IR	3	0.5 (Transmittance)	[21]
Hybrid plasmonic metasurface	Plasmonic	Mid-IR	12	0.8 (Reflectance)	[22]
**Dielectric thermally tunable metasurface**	**quasi-BIC**	**Mid-IR**	**200**	**0.5 (Reflectance)**	This work

Table S1: Literature comparison on VO_2 metasurfaces. Comparative overview of VO_2 metasurfaces based on resonance type, operational spectral range, experimental Q-factor, and absolute switching contrast. The experimental Q-factor and absolute switching contrast were extracted from published plots, therefore, small deviations between the stated and actual values may be present.

SUPPORTING FIGURES

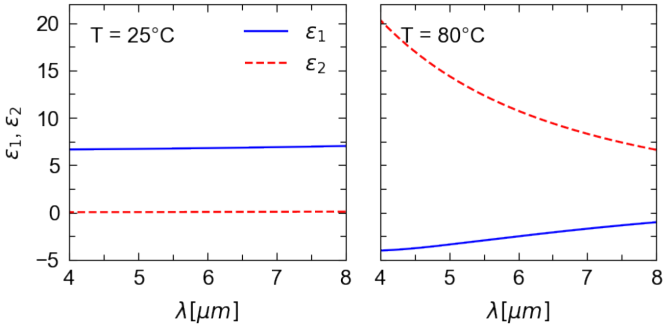

Figure S1: VO_2 ellipsometry. Ellipsometry data between 4 and 8 μm for the used 30 nm VO_2 film measured at 25°C for the cold phase and at 80°C for the hot phase. Blue solid (red dashed) lines represent thereal (imaginary) part $\varepsilon_1(\varepsilon_2)$ of the VO_2 dielectric function.

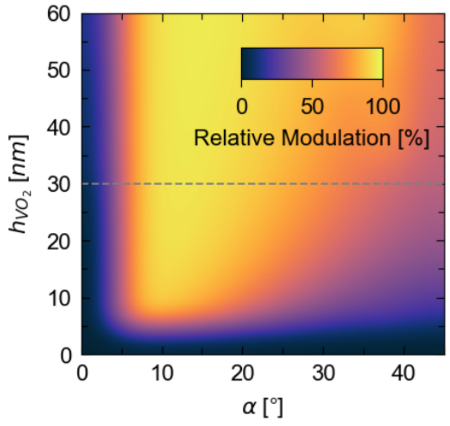

Figure S2: Numerical Relative Reflectance Modulation. VO_2 layer height and asymmetry α sweep with the relative reflectance modulation max$\left(\frac{R_{\text{cold}}-R_{\text{hot}}}{R_{\text{cold}}}\right)$ plotted in color. The maximal value is 97%.

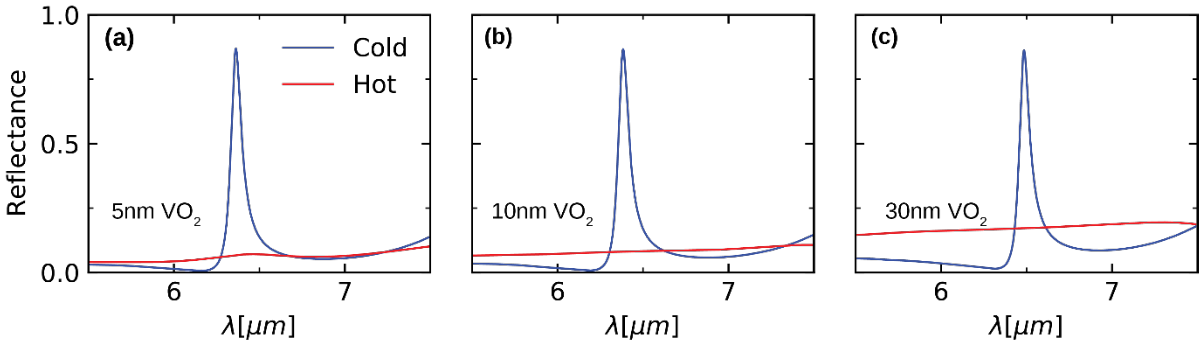

Figure S3: Numerical reflectance spectra for continuous VO_2 films. The same structural parameters were used as in Figure 2 with α=20°, but with a continuous VO_2 layer just below the resonators and above the substrate, without a VO_2 layer on top of the resonators and no SiO_2 capping layer. In (a), the VO_2 layer thickness is 5 nm, in (b) 10 nm, and in (c) 30 nm. The switching performance, visible from the shown reflectance spectra in the cold and hot phases, is high for all

layer thicknesses. Nevertheless, due to the continuous films, off-resonance switching is observable, especially for thicknesses of 10 nm and 30 nm.

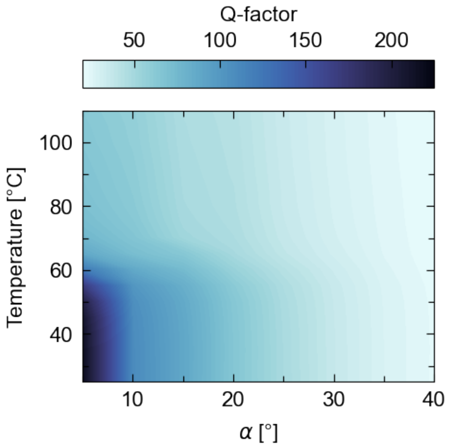

Figure S4: Q-factor mapping. Total fitted Q-factor according to our TCMT model, calculated from the two loss rates γ_{rad} and γ_{int} by $Q = \omega_0(\gamma_{\mathrm{rad}} + \gamma_{\mathrm{int}})^{-1}$.

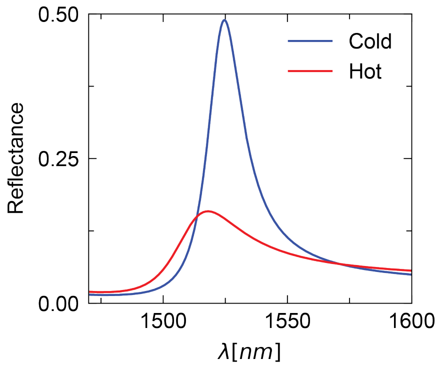

Figure S5: Numerical reflectance spectra in the near-IR. The geometry depicted in Figure 2 was uniformly scaled down by multiplying all geometrical parameters by a factor of 0.23, while maintaining a VO_2 layer thickness of 20 nm and an ellipse tilt angle of 20°. The permittivities used were $\varepsilon_1 = 9.24$ and $\varepsilon_2 = 2.47$ in the cold phase, and $\varepsilon_1 = -2.60$ and $\varepsilon_2 = 10.45$ in the hot phase. These permittivity values were determined via ellipsometry.

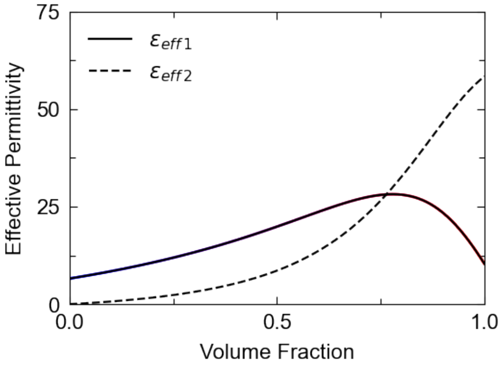

Figure S6: Effective Permittivity. Effective permittivity of VO_2 for different volume fractions VF, using k and n values at a wavelength of 6.5 μm.

SUPPORTING REFERENCES

1. Voloshenko, I. *et al.* Microscopic nature of the asymmetric hysteresis in the insulator-metal transition of VO2 revealed by spectroscopic ellipsometry. *Appl. Phys. Lett.* **113**, (2018).

2. Liu, B., Gong, M., Zhang, J., Tian, S. & Zhao, X. Comparative study of the metal insulator transition of a VO2film with simultaneous infrared thermography and electric measurements. *AIP Adv.* **11**, (2021).

3. Leahu, G., Li Voti, R., Sibilia, C. & Bertolotti, M. Anomalous optical switching and thermal hysteresis during semiconductor-metal phase transition of VO2 films on Si substrate. *Appl. Phys. Lett.* **103**, (2013).

4. Fan, S., Suh, W. & Joannopoulos, J. D. Temporal coupled-mode theory for the Fano resonance in optical resonators. *J. Opt. Soc. Am. A* **20**, 569 (2003).

5. Tripathi, A. *et al.* Tunable Mie-Resonant Dielectric Metasurfaces Based on VO2Phase-Transition Materials. *ACS Photonics* **8**, 1206–1213 (2021).

6. Kang, T. *et al.* Mid-infrared active metasurface based on Si/VO 2 hybrid meta-atoms . *Photonics Res.* **10**, 373 (2022).

7. Zhang, W., Wu, X., Li, L., Zou, C. & Chen, Y. Fabrication of a VO2-Based Tunable Metasurface by Electric-Field Scanning Probe Lithography with Precise Depth Control. *ACS Appl. Mater. Interfaces* **15**, 13517–13525 (2023).

8. King, J. *et al.* Electrically tunable VO2–metal metasurface for mid-infrared switching, limiting and nonlinear isolation. *Nat. Photonics* **18**, 74–80 (2024).

9. Liu, L., Kang, L., Mayer, T. S. & Werner, D. H. Hybrid metamaterials for electrically triggered multifunctional control. *Nat. Commun.* **7**, 1–8 (2016).
